# NbALY916 is involved in potato virus X P25‐triggered cell death in *Nicotiana benthamiana*

**DOI:** 10.1111/mpp.12986

**Published:** 2020-09-06

**Authors:** Xue Yang, Yanzhen Tian, Xing Zhao, Liangliang Jiang, Ying Chen, Shuzhen Hu, Stuart MacFarlane, Jianping Chen, Yuwen Lu, Fei Yan

**Affiliations:** ^1^ State Key Laboratory for Managing Biotic and Chemical Threats to the Quality and Safety of Agro‐products Key Laboratory of Biotechnology in Plant Protection of Ministry of Agriculture and Zhejiang Province Institute of Plant Virology Ningbo University Ningbo China; ^2^ College of Plant Protection Henan Agriculture University Zhengzhou China; ^3^ College of Plant Protection Fujian Agriculture and Forestry University Fuzhou China; ^4^ The James Hutton Institute, Cell and Molecular Sciences Group Invergowrie, Dundee UK

**Keywords:** cell death, H_2_O_2_, NbALY916, P25, *potato virus X*

## Abstract

Systemic necrosis often occurs during viral infection of plants and is thought mainly to be the result of long‐term stress induced by viral infection. Potato virus X (PVX) encodes the P25 pathogenicity factor that triggers a necrotic reaction during PVX‐potato virus Ysynergistic coinfection. In this study, we discovered that NbALY916, a multifunctional nuclear protein, could interact with P25. When NbALY916 expression was reduced by tobacco rattle virus (TRV)‐based virus‐induced gene silencing, the accumulation of P25 was increased, which would be expected to cause more severe necrosis. However, silencing of *NbALY916* reduced the extent of cell death caused by P25. Furthermore, we found that overexpression of NbALY916 increased the accumulation of H_2_O_2_ and triggered more extensive cell death when coexpressed with P25, even though accumulation of P25 was itself reduced by the increased expression of NbALY916. Furthermore, transient expression of P25 specifically induced the expression of *NbALY916* mRNA, but not the mRNAs of three other ALYs in *Nicotiana benthamiana*. In addition, we showed that silencing of NbALY916 or transient overexpression of NbALY916 affected the infection of PVX in *N. benthamiana*. Our results reveal that NbALY916 has an antiviral role that, in the case of PVX, operates by inducing the accumulation of H_2_O_2_ and mediating the degradation of P25.

## Graphical abstract

Nuclear protein NbALY916, which is involved in the process of potato virus X P25‐induced hypersensitive response‐like cell death, can interact with P25 and mediate its degradation, indicating an antiviral role of NbALY916 during PVX infection of *Nicotiana benthamiana*.

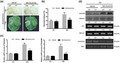

ALY proteins (Yar1 in yeast, Aly/RNA export factor [REF] in metazoans) are known to be recruited to mRNAs to facilitate their export from the nucleus (Cullen, 2003; Katahira *et al*., 2009; Pfaff *et al*., 2018). There are four ALY proteins in *Arabidopsis thaliana*, among which ALY1 has been identified to transport the mRNA of the RNA‐directed DNA methylation (RdDM) factor *ARGONAUTE6* out of the nucleus (Choudury *et al*., 2019). In mammalian cells, Aly/REF is involved in aiding nuclear export of viral RNA by directly interacting with viral proteins (Balasubramaniam *et al*., 2013; Tian *et al*., 2013). In plants, tomato bushy stunt virus (TBSV) P19 can interact with ALY proteins and relocalize two of them from the nucleus to the cytoplasm (Uhrig *et al*., 2004). The two other ALY proteins repress the silencing suppressor activity of P19 by altering its localization from the cytoplasm into the nucleus (Canto *et al*., 2006).

Potato virus X (PVX), a positive‐sense single‐stranded RNA virus, belongs to the genus *Potexvirus* and its genome carries a block of three partially overlapping open reading frames termed the “triple gene block” (TGB) that encode proteins required for virus cell‐to‐cell movement, among which TGBp1 (termed P25) is a multifunctional protein that acts as a viral suppressor of RNA silencing (VSR) and participates in organization of the viral replication complex (VRC) (Chiu *et al*., 2010; Tilsner *et al*., 2012). Furthermore, P25 is a pathogenicity factor that triggers cell death in PVX‐associated synergisms and also is a putative avirulence (avr) protein inducing plant hypersensitive response (HR)‐like cell death in a threshold‐dependent manner (Aguilar *et al*., 2015, 2018). Gonzalez‐Jara and colleagues found that the enhancement of pathogenicity associated with the synergistic interaction of PVX and plum pox virus (PPV) is not a consequence of more efficient PVX replication due to RNA silencing suppression by the PPV VSR, HC‐Pro (Gonzalez‐Jara *et al*., 2005), but was related to the accumulation level of the PVX P25 protein. In our experiments, we found that transient expression of P25 using a high concentration of agrobacteria triggered an HR‐like response at 9 days postinfiltration (dpi) in *Nicotiana benthamiana* leaves, whereas treatment with the same concentration of agrobacteria but lacking the P25 expression plasmid did not induce an HR (Figure S1). The unfolded protein response (UPR) was shown to contribute to cell death induced by P25‐associated PVX/PPV synergism (Aguilar *et al*., 2018), but Ye *et al*. found that agroexpression of PVX P25 was a much weaker inducer of UPR‐related genes than PVX TGBp3 (Ye *et al*., 2011). Hence, it was hypothesized that P25 might trigger HR by other non‐UPR‐related processes.

To identify potential host proteins that interact with the PVX P25 or participate in P25‐mediated HR, P25 fused at the C‐terminus to green fluorescent protein (GFP) was transiently expressed in *N. benthamiana* leaves. Putative plant P25‐interacting proteins were collected by coimmunoprecipitation (Co‐IP) with GFP‐binding magnetic beads and identified by mass spectrometry (MS) (Figure S2). MS results revealed 98 plant proteins that potentially interacted with P25 (Table. S1). Among them was Hin19 (Q6JAC7), called NbALY916 (AM167906.1) in *N. benthamiana*, which was shown to be involved in Nep1_Mo_ (a Nep1‐like protein from *Magnaporthe oryzae*)‐triggered hypersensitive cell death (Teng *et al*., 2014).

To confirm the P25–NbALY916 interaction, we first performed bimolecular fluorescence complementation (BiFC) experiments by infiltration of agrobacterial cultures carrying various expression vectors into nuclear‐labelled *N. benthamiana* leaves (H2B‐red fluorescent protein [RFP] transgenic plants). NbALY916 and P25 were fused to the C‐terminus of either the N‐proximal or C‐proximal half of yellow fluorescent protein (YFP) to generate NbALY916‐nYFP, P25‐cYFP, and P25‐nYFP, together with β‐glucuronidase (GUS)‐cYFP and GUS‐nYFP as noninteracting control constructs. Coexpression of NbALY916‐nYFP and P25‐cYFP resulted in YFP fluorescence signals in the nucleus of agroinfiltrated cells at 72 hr postinfiltration (hpi). Meanwhile, the self‐interaction of P25 (P25‐nYFP + P25‐cYFP) was used as a positive interaction control. Combinations of P25‐nYFP/GUS‐cYFP, NbALY916‐nYFP/GUS‐cYFP, P25‐cYFP/GUS‐nYFP, and GUS‐nYFP/GUS‐cYFP failed to fluoresce, demonstrating that neither NbALY916 nor P25 produced nonspecific fluorescence (Figure 1a). These results indicated that P25 interacts with NbALY916 in the nucleus in BiFC assays. Co‐IP was then performed to verify the interaction between NbALY916 and P25. In this assay, plasmids encoding NbALY916‐GFP and P25 were transiently expressed in *N. benthamiana*, with free GFP being expressed as a noninteracting control protein, and using GFP‐TRAP_M beads to collect GFP and GFP‐tagged proteins from the infiltrated leaf extract. P25 was precipitated in the presence of NbALY916‐GFP but not with free GFP or when expressed alone in the plant (Figure 1b).

**FIGURE 1:**
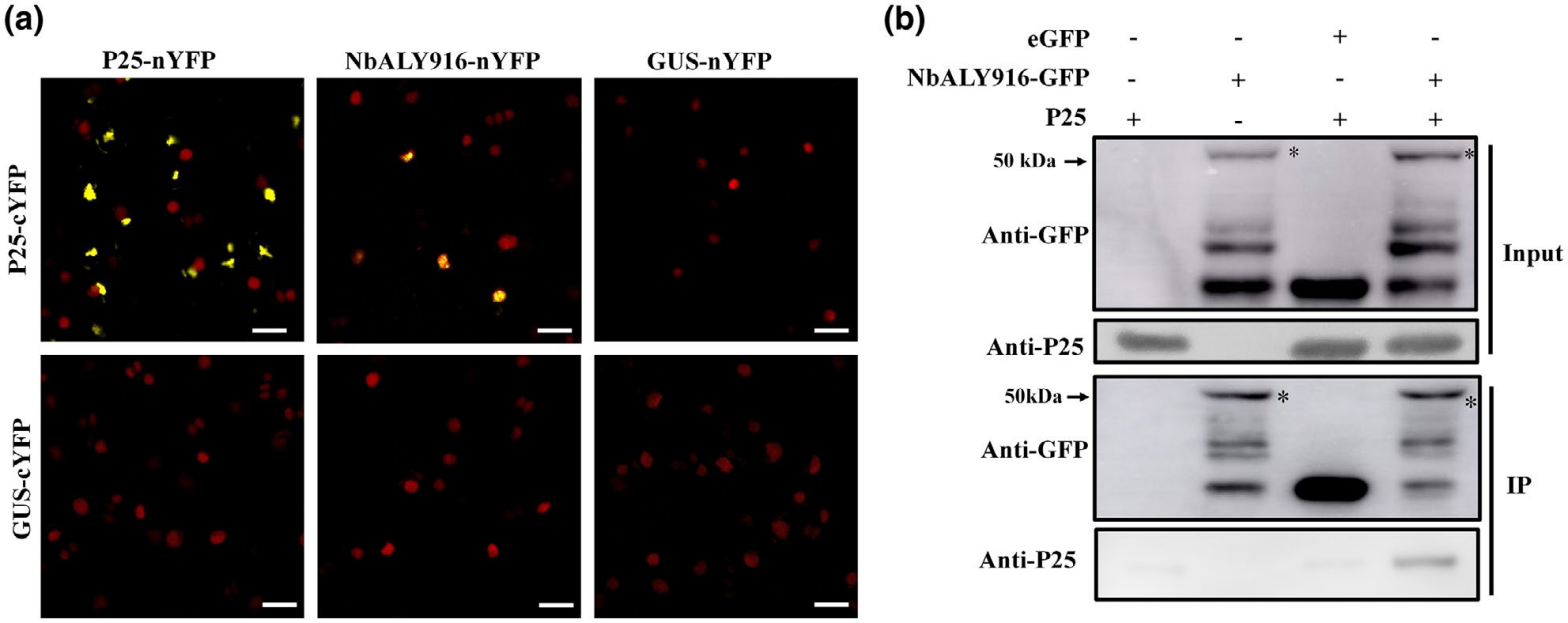
PVX P25 interacts with NbALY916. (a) Interaction between NbALY916 and P25 revealed by bimolecular fluorescence complementation in agrofiltrated *Nicotiana benthamiana* leaves. Bars, 20 μm. The self‐interaction of P25 was used as a positive control and β‐glucuronidase (GUS) was used as a noninteracting control. (b) Interaction between NbALY916 and P25 verified by coimmunoprecipitation. The input proteins were detected by western blotting with green fluorescent protein (anti‐GFP) and anti‐P25 antibodies. IP denotes the immunoprecipitated protein fraction probed with anti‐GFP and anti‐P25 antibodies. The asterisks indicating bands of NbALY916‐GFP in western blots. The multiple bands detected by the anti‐GFP antibody are most probably nonspecific cleavage products of the NbALY916‐GFP fusion protein

It was reported that silencing of *NbALY916* by tobacco rattle virus (TRV)‐based virus‐induced gene silencing (VIGS) compromised the cell death triggered by Nep1_Mo_ in *N. benthamiana* (Teng *et al*., 2014). To test whether NbALY916 is also involved in the P25‐induced HR, we similarly used TRV VIGS to silence *NbALY916* in *N. benthamiana* plants before infiltrating them with P25. At 10 dpi, the *NbALY916‐*silenced plants (TRV:NbALY916) showed no obvious change in appearance when compared with wild‐type TRV (TRV:00; empty vector) as a control treatment (Figure S3a). Quantitative reverse transcription PCR (RT‐qPCR) results showed that the transcript level of *NbALY916* in TRV:NbALY916‐silenced plants was only 4% of that in TRV:00‐treated plants; meanwhile, silencing of *NbALY916* had no effect on the expression of three other ALY genes, *NbALY1693*, *NbALY615*, and *NbALY617* (Figure S3b). At 10 dpi of the VIGS experiments, we expressed the P25 protein transiently by a second agrobacterial infiltration into the systemically infected leaves of the same plants, including an empty expression vector (EV) as a negative control treatment in a separate area of the same leaves. Seven days after P25 infiltration, we used 3,3′‐diaminobenzidine (DAB) staining to show the accumulation of hydrogen peroxide (H_2_O_2_) in the infiltrated areas (Figure 2a, upper panels) and measured electrolyte leakage to assay cell damage (Figure 2b) (Aguilar *et al*., 2018). The results showed that the accumulation of H_2_O_2_ and percentage of electrolyte leakage in P25‐infiltrated patches in TRV:NbALY916‐silenced leaves was less than that in P25‐infiltrated patches in nonsilenced leaves. At 9 dpi, we could observe cell death in P25‐containing patches in TRV:00‐treated leaves, but the cell death triggered by P25 was compromised in the TRV:NbALY916‐treated leaves (Figure 2a, bottom panels). These results indicate that silencing *NbALY916* inhibited cell death caused by P25 expression. We used RT‐qPCR to detect the expression levels of *NbrbohA* and *NbrbohB*, which encode plant NADPH oxidases and are responsible for generating the H_2_O_2_ involved in the HR (Yoshioka *et al*., 2003). In our experiments, the expression of *NbrbohA* and *NbrbohB* induced by P25 expression was significantly decreased in *NbALY916‐*silenced leaves compared with TRV:00‐treated leaves (Figure 2c).

**FIGURE 2:**
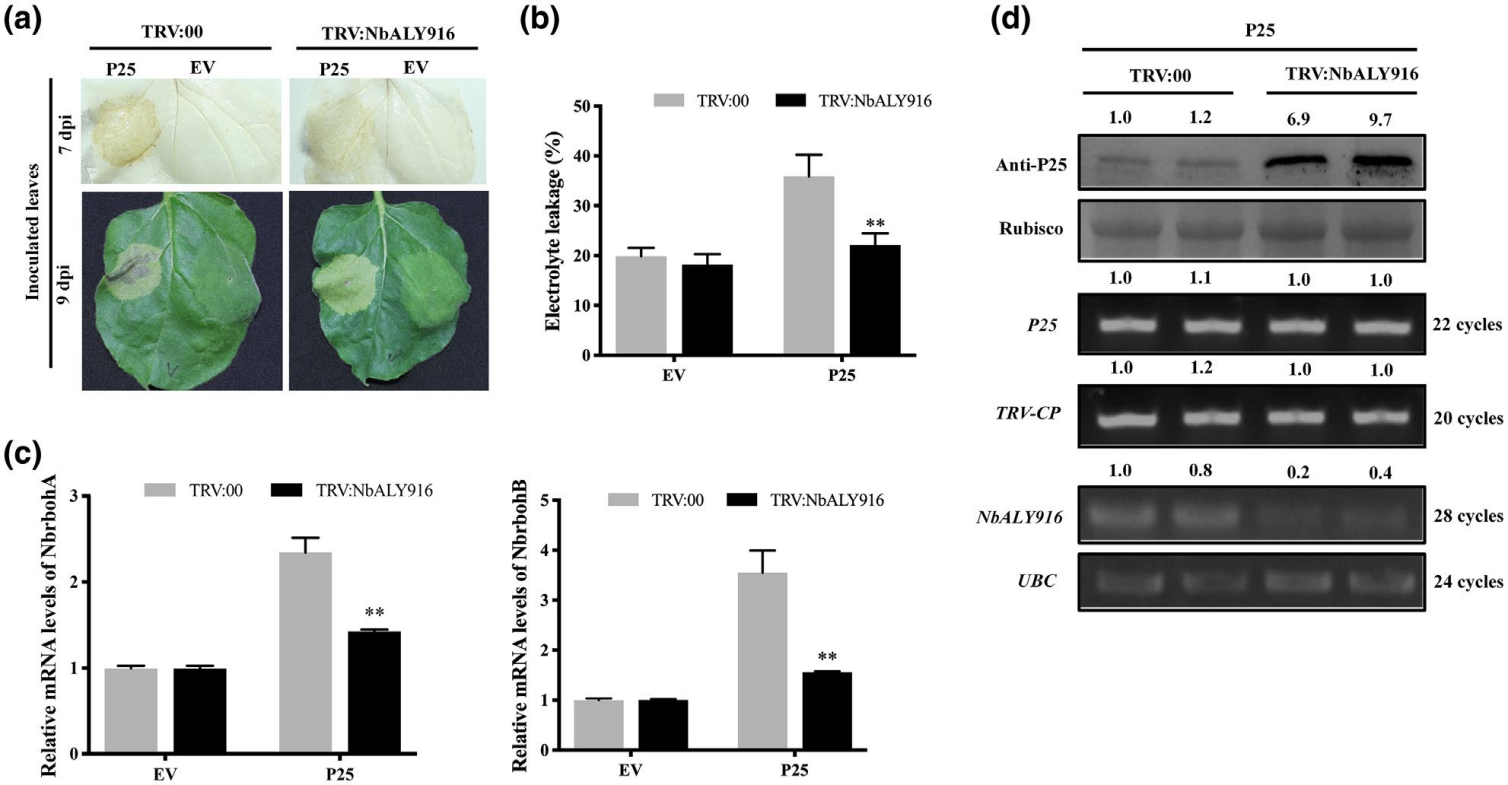
Silencing of *NbALY916* inhibits H_2_O_2_ accumulation and cell death induced by P25. (a) Upper panel, 3,3′‐diaminobenzidine (DAB) staining (7 days postinoculation, dpi) of P25 (OD_600_ = 0.1) or empty vector (EV, OD_600_ = 0.1) infiltrated into leaves of TRV:00‐ and TRV:NbALY916‐treated plants. The lower panel, visible necrosis in similarly treated leaves at 9 dpi. (b) Leaf discs were excised and assayed for electrolyte leakage at 9 dpi. Bars represent the standard errors of the means from three biological repeats, each consisting of six plants. Error bars show SD and the graph represents the combined data from three independent replicates. A two‐sample unequal variance directional t test was used to test the significance of the difference (**p < .01). (c) Quantitative reverse transcription PCR quantification of NbrbohA and NbrbohB mRNAs in TRV:00 and TRV:NbALY916 plants after treatment with P25 or EV. Primer sequences are listed in Table S2. Bars represent the SEM from three biological repeats. A two‐sample unequal variance directional t test was used to test the significance of the difference (**p < .01). (d) Western blotting and reverse transcription PCR detection of the P25 protein and P25, TRV‐CP and NbALY916 mRNAs in NbALY916‐silenced and nonsilenced (TRV:00) leaves. The level of P25 protein accumulation was calculated relative to that of RuBisCO and the transcription levels of P25, TRV CP and NbALY916 were normalized relative to UBC (Ubiquitin C). The relative protein and mRNA levels were calculated using ImageJ

Western blotting was performed at 5 dpi to detect the accumulation of the P25 protein, showing that in TRV:NbALY916‐treated leaves P25 accumulation was higher than in TRV:00‐treated leaves. Semiquantitative PCR results showed that the mRNA levels of P25 and TRV coat protein (CP) were not affected and *NbALY916* was indeed silenced in TRV:NbALY916‐treated leaves (Figure 2d). The increased level of P25 should cause more severe necrosis in *NbALY916‐*silenced plants, but the extent of cell death was reduced in our experiments (Figure 2a). Hence, we hypothesized that the expression level of *NbALY916* was one of the factors that affects the cell death triggered by expression of P25.

Overexpression of NbALY916 fused to GFP (NbALY916‐GFP) induced the accumulation of H_2_O_2_ and cell death (Figure 3a,b), and unfused NbALY916 had the same effects (Figure S4). Also, RT‐qPCR analysis showed that the mRNA level of *NbrbohA* was up‐regulated by expression of NbALY916‐GFP, but not by free GFP (Figure 3c). The mRNA levels of *NbALY916* were increased at 3, 5, 7, and 9 dpi in patches infiltrated with P25 compared with control leaves (Figure S5a), but this was not the case with the other *ALY* mRNAs, *NbALY617* and *NbALY1693*/*NbALY615* (Figure S5b,c).

**FIGURE 3:**
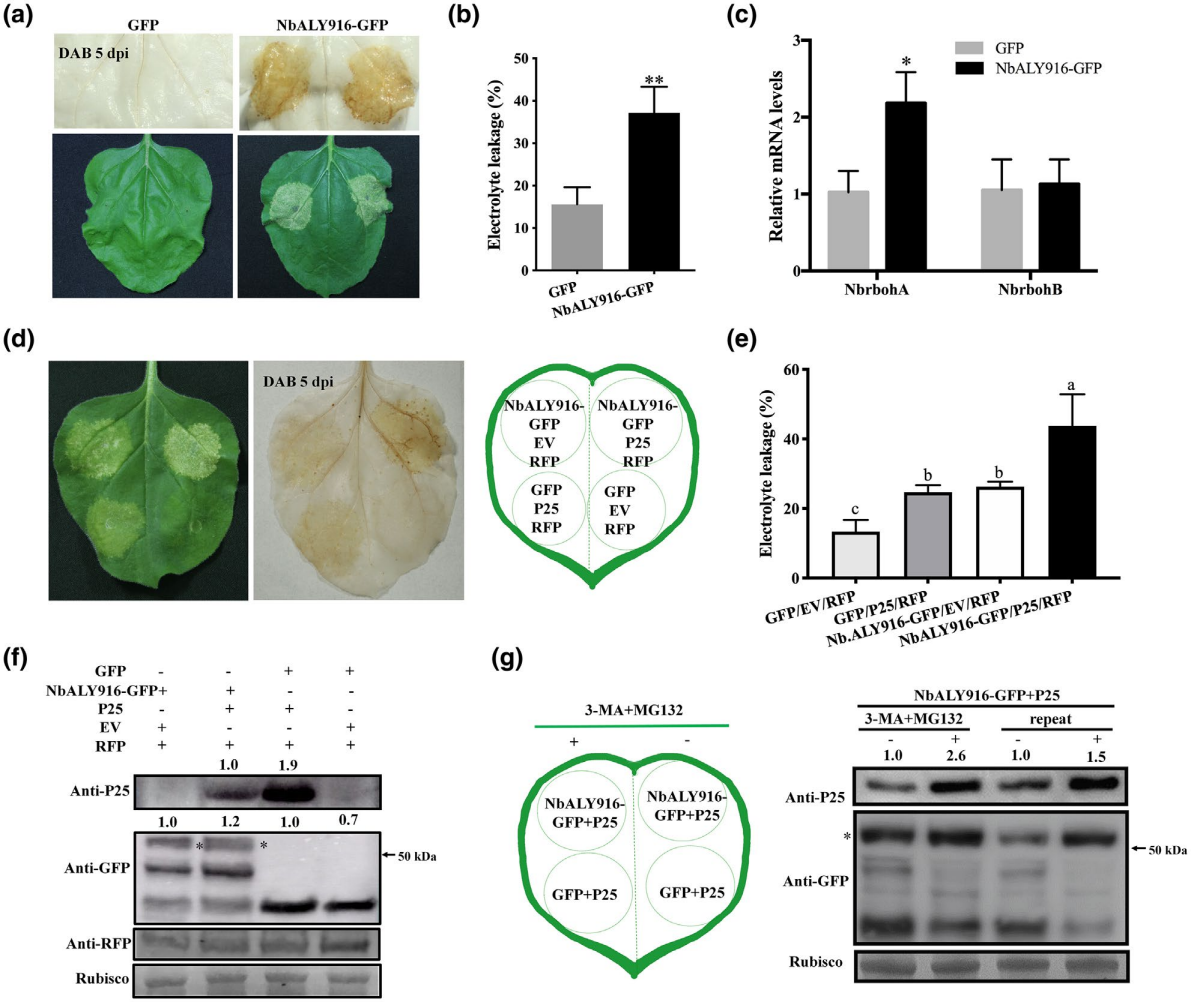
Overexpression of NbALY916 promotes H_2_O_2_ accumulation and cell death induced by P25. (a) The accumulation of H_2_O_2_ (upper panel, 3,3′‐diaminobenzidine [DAB] stained) and cell death (lower panel, visual symptoms) observed after transient expression of NbALY916‐GFP or free green fluorescent protein (GFP) in leaves at 5 days postinoculation (dpi). (b) Leaf discs were excised and assayed for electrolyte leakage at 5 dpi. Bars represent the *SEM* from three biological repeats, each consisting of six plants. A two‐sample unequal variance directional *t* test was used to test the significance of the difference (**p < .01). (c) The transcript levels of *NbrbohA* and *NbrbohB* after transient expression of NbALY916‐GFP or free GFP. Bars represent the *SEM* from three biological repeats. A two‐sample unequal variance directional *t* test was used to test the significance of the difference (*p < .05). (d) Visible necrosis (left panel) and H_2_O_2_ accumulation (right panel, DAB stained) after transient expression of combinations of P25, NbALY916‐GFP, empty vector (EV), and free GFP. The expression of red fluorescent protein (RFP) was used as the reference of heterogeneous expression. (e) Leaf discs were excised and assayed for electrolyte leakage at 5 dpi. Bars represent the *SEM* from three biological repeats, each consisting of six plants. Error bars show *SD* and the graph represents the combined data from three independent replicates. Letters on the graph denote statistically significant differences (analysis of variance, *p* ≤ .05). (f) Western blotting detection of P25, NbALY916, and RFP in infiltrated leaves mentioned in (c). The value of P25 protein accumulation was normalized to RuBisCO. The indicated bands of NbALY916‐GFP were used to calculate the protein accumulation levels by ImageJ. (g) P25 coexpressed with ALY916‐GFP or GFP in the same leaf for 48 hr, followed by 10 mM 3‐methyladenine (3‐MA) and 100 μM MG132 for 4 hr. Western blotting detection of P25 and NbALY916 expressed with (+; 4 hr postinfiltration) or without (−) the addition of a mixture of 10 mM 3‐MA and 100 µM MG132. The P25 protein accumulation was normalized to RuBisCO and the relative protein levels were calculated in relation to the 1% dimethyl sulphoxide‐only treatment. The relative protein levels were calculated by ImageJ. The asterisks indicate bands of NbALY916‐GFP in western blots

We next investigated whether the extent of cell death was enhanced or not when P25 was coexpressed with NbALY916. We found at 5 dpi that coexpression of NbALY916‐GFP and P25 triggered stronger cell death than either of the proteins expressed singly, and the DAB staining and electrolyte leakage assay results showed that H_2_O_2_ and percentage of electrolyte leakage were the highest in leaves where NbALY916‐GFP and P25 were expressed together (Figure 3d,e). In addition, the protein level of P25, when coexpressed with NbALY916‐GFP, was lower than when P25 was coexpressed with free GFP. In these experiments transient expression of RFP was used as a control for agroinfiltration effects and RuBisCO was used as a reference for the stability of endogenous leaf proteins (Figure 3f). The results of these experiments suggest that coexpression with NbALY916 reduces the accumulation of P25 and that reducing the level of NbALY916 (by silencing) allows increased amounts of P25 to accumulate, perhaps by reducing degradation of P25.

The main protein degradative routes in eukaryotes are the ubiquitin‐26S proteasome system (UPS) and autophagy (Varshavsky, 2017). To elucidate which pathway was responsible for the degradation of the P25 protein when coexpressed with NbALY916, further coinfiltrations were performed with the addition of either MG132 (an inhibitor of the 26S proteasome pathway) or 3‐methyladenine (3‐MA, an inhibitor of autophagy) (Seglen and Gordon, 1982; Tanida *et al*., 2005). Western blotting showed that the accumulation of P25 when coexpressed with NbALY916 was not affected by treatment with MG132 or 3‐MA (Figure S6a). It was previously shown that autophagy could impact on the 26S proteasome's degradative route and remove excess or damaged proteasomes when the proteasome was inhibited by chemical or genetic deficiency (Marshall *et al*., 2015), which indicated that UPS and autophagy could complement one another to manage the recycling of nutrients and mitigate proteotoxic stress. Therefore, we decided to combine both 3‐MA and MG132 treatments and then examine the accumulation of the P25 protein coexpressed with NbALY916‐GFP or free GFP in the same leaves (Figure 3g). The accumulation levels of P25 in P25 and NbALY916 coexpressed patches were increased in the treatment of MG132 plus 3‐MA compared with the mock treatment (Figure 3g). The treatment of MG132 plus 3‐MA did not significantly affect P25 accumulation in combination with free GFP (Figure S6b). However, the degradative routes by which P25 is repressed via NbALY916 requires further investigation. The results above suggest that the transcription level of *NbALY916* was specifically induced by expression of P25, while increased expression of NbALY916 further mediated the degradation of P25. Coexpression of P25 and NbALY916 induced severe necrosis, but with low accumulation of P25, and silencing of NbALY916 compromised the necrosis induced by P25. Combined with the finding that overexpression of NbALY916 induced necrosis, we suggest that the accumulation level of NbALY916 plays an important role in the induction of necrosis by P25.

In consideration of the relationship between NbALY916 and P25, we next wanted to investigate the role of NbALY916 in the process of PVX infection. We rub‐inoculated PVX‐GFP onto leaves of plants that had been treated 10 days previously with TRV:NbALY916 (to induce *NbALY916* silencing) or TRV:00 (as a nonsilenced control). The progress of infection in the inoculated and upper noninoculated leaves was monitored with a UV lamp to observe the location and extent of GFP fluorescence produced by PVX‐GFP. Although all plants were systemically infected by PVX‐GFP at 5 dpi, the rate of infection in TRV:NbALY916‐treated plants developed faster than in TRV:00 treated plants (Figure 4a). The foci of GFP fluorescence in inoculated leaves of TRV:NbALY916‐treated plants were similar to those in TRV:00 treated plants. Similarly, western blotting and northern blotting results showed no obvious differences in the accumulation of PVX CP and PVX RNA levels in the mechanically inoculated leaves of TRV:NbALY916‐ and TRV:00‐treated plants (Figure 4b, upper lanes,c). However, in systemically infected leaves, the GFP fluorescence in *NbALY916‐*silenced plants was brighter than that in TRV:00‐treated plants at 6 dpi (Figure 4b, bottom lanes). Similarly, western blotting and northern botting results showed higher levels of PVX CP and viral RNA in the upper leaves of *NbALY916‐*silenced leaves compared to nonsilenced plants (Figure 4d). We then coexpressed NbALY916‐GFP and PVX, and used free GFP and PVX as a control in the same leaf (Figure 4e). At 3 dpi, we used western blotting to detect the accumulation of PVX CP. The results showed that transient expression of NbALY916‐GFP reduced the accumulation of PVX CP compared with transient expression of free GFP (Figure 4f). Taken together, these results suggest that NbALY916 plays an antiviral role during the infection of PVX in *N. benthamiana*.

**FIGURE 4:**
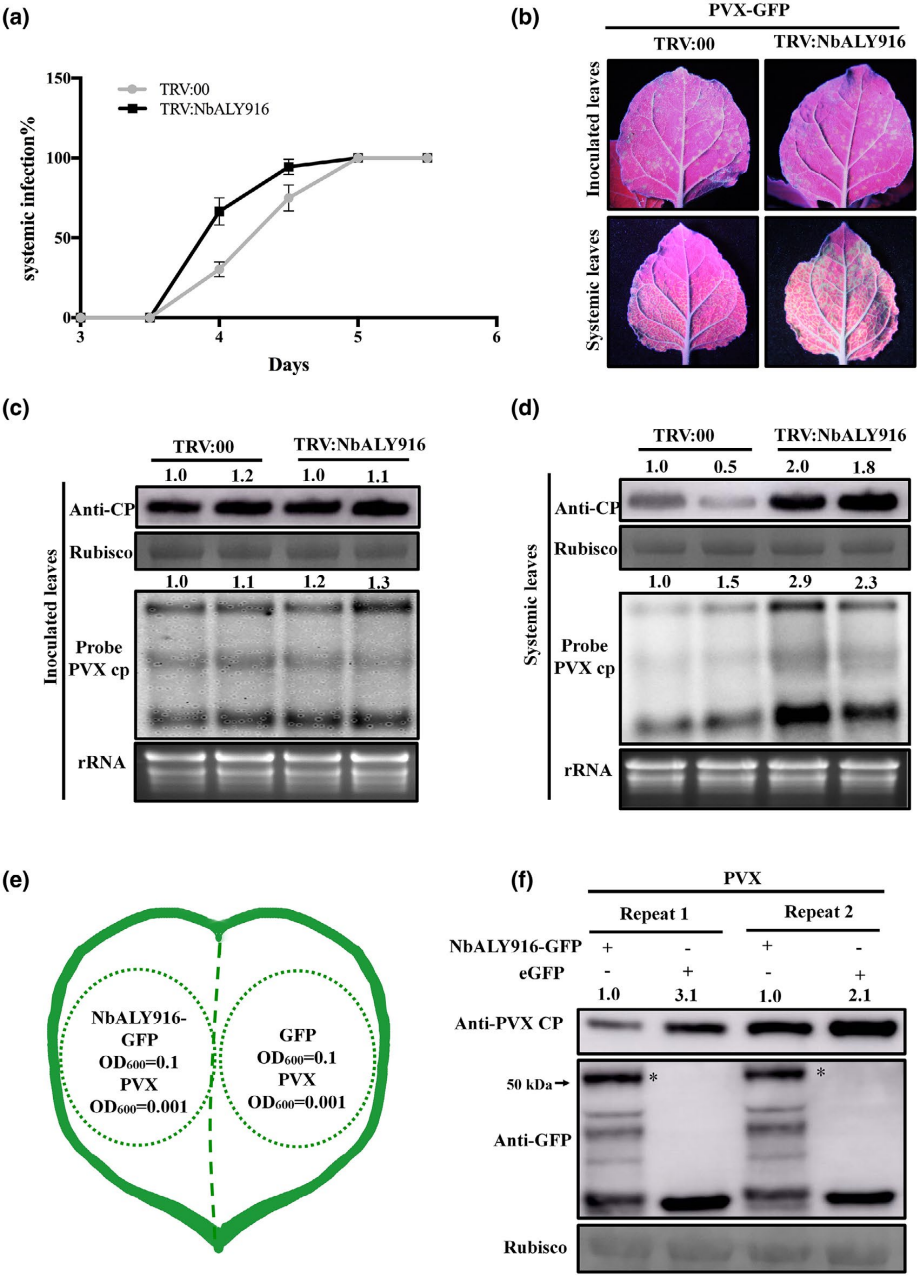
NbALY916 affects PVX infection. (a) Time‐course analysis of PVX‐green fluorescent protein (GFP) infection into upper (systemically infected) leaves of TRV:00‐ and TRV:NbALY916‐treated plants. Error bars represent the *SD* for three independent experiments each using 15 plants per treatment. (b) Appearance of green fluorescent foci of PVX‐GFP infection, photographed under UV light at 3 days postinoculation (dpi) (inoculated leaves) and 6 dpi (systemically infected leaves). (c) and (d) PVX coat protein (CP) and viral RNA accumulation in inoculated (at 3 dpi) and systemically infected (at 6 dpi) leaves of TRV:00‐ and TRV:NbALY916‐treated plants, detected by western blotting and northern blotting. The CP and viral RNA accumulation were normalized to RuBisCO and rRNA, respectively, and the relative levels were calculated in relation to TRV:00 treatment. The relative protein and RNA levels (using all viral RNA from PVX and rRNA) were calculated using ImageJ. (e) Agroinfiltrated PVX (OD_600_ = 0.001) with NbALY916‐GFP (OD_600_ = 0.1) or GFP (OD_600_ = 0.1) on the same leaf. (F) PVX CP and NbALY916‐GFP accumulation in the same leaf (at 3 dpi) detected by western blotting. The CP and NbALY916‐GFP accumulation were detected using anti‐PVX CP and anti‐GFP antibodies, respectively, and the relative levels were calculated in relation to coexpressed PVX and GFP using ImageJ. The asterisks indicating bands of NbALY916‐GFP in western blots

HR‐conferred resistance to viral infection restricts virus spread and is accompanied by the induction of rapid cell death (Kombrink and Schmelzer, 2001). In some viral infections, the HR is initiated by Avr/R protein interactions. For example, the tomato R protein Sw‐5b recognizes a conserved epitope in the tospovirus NSm protein and triggers the HR to confer broad‐spectrum resistance (Zhu *et al*., 2017). Cell death or systemic necrosis is also a feature of the plant response in many compatible viral infections. It is reported that the systemic necrosis caused by PVX‐associated synergisms is a threshold‐dependent immune response induced by P25 (Aguilar *et al*., 2015). Silencing of the host genes *SGT1* and *RAR1*, or overexpression of the endoplasmic reticulum luminal binding protein (BiP), alleviated the HR (Aguilar *et al*., 2015, 2018). In our study, we identified a new host nuclear protein NbALY916 that is involved in the P25‐triggered HR. Furthermore, we revealed that transient overexpression of NbALY916 in *N. benthamiana* could induce cell death accompanied by the accumulation of H_2_O_2_ and electrolyte leakage. Our results linking NbALY916 with the plant reaction to virus infection are consistent with an earlier study in which silencing of *NbALY916* inhibited the accumulation of H_2_O_2_ that normally occurs after treatment with the fungal Nep1_Mo_ cell death elicitor (Teng *et al*., 2014). Our observation that NbALY916 is also involved in the turnover of P25 by an as yet undiscovered mechanism suggests there could be a common pathway for NbALY916 interaction with pathogenicity elicitors from various plant pathogens as part of basal HR‐associated plant defence.

## Supporting information


**FIGURE S1** PVX P25 induces the accumulation of H_2_O_2_ and cell death. (a) Visible necrosis (centre image) and H_2_O_2 _accumulation (right image; DAB stained) in *Nicotiana benthamiana* leaf patches after infiltration with different concentrations (OD_600_) of agrobacteria harbouring a P25 expression plasmid or empty vector (EV). (b) Western blotting detection of P25 protein in infiltrated patches. The P25 protein accumulation was normalized to RuBisCO and the relative levels were calculated in relation to EV treatment. The relative protein levels were calculated using ImageJ. (c) Leaf discs were excised and assayed for electrolyte leakage. Bars represent the standard errors of the means from three biological repeats, each consisting of six plants. Error bars show *SD* and the graph represents the combined data from three independent replicates. Letters on the graph denote statistically significant differences (ANOVA, *p* ≤ .05)

**FIGURE S2** Proteins precipitated with transiently expressed green fluorescent protein (GFP) and P25‐GFP by GFP beads. SDS‐PAGE results of precipitated proteins by GFP beads. The GFP lane shows transiently expressing GFP and then precipitation with GFP beads. P25‐GFP lane shows transiently expressing P25‐GFP and then precipitation with GFP beads. The gels in the red boxes were sent for mass spectrum (MS) identification

**FIGURE S3** Phenotype of TRV‐induced *NbALY916* silencing on *Nicotiana benthamiana* at 10 days postinoculation (dpi). (a) Comparison of infection symptoms caused by TRV:00 and TRV:NbALY916 at 10 dpi. (b) The silencing efficiency of TRV:NbALY916 was measured by quantitative reverse transcription PCR. Primer sequences are listed in Table S2. Bars represent the *SEM* from three biological repeats. A two‐sample unequal variance directional *t* test was used to test the significance of the difference (**p < .01)

**FIGURE S4** Transient overexpression of NbALY916 and NbALY916‐GFP induces cell death. The accumulation of H_2_O_2 _(upper panel, DAB stained) and cell death (lower panel, visual symptoms) observed after transient expression of NbALY916‐GFP and NbALY916‐GFP at 5 days postinoculation

**FIGURE S5** P25 induces the expression of *NbALY916*. Results of quantitative reverse transcription PCR to measure the mRNA levels of *NbALY916* (a), *NbALY1693* and *615* (b) and *NbALY617* (c) in leaves infiltrated with P25 or empty vector (EV). Bars represent the *SEM* from three biological repeats. A two‐sample unequal variance directional *t* test was used to test the significance of the difference (*p < .05; **p < .01)

**FIGURE S6** The accumulation of P25 with chemical treatment. (a) Western blotting showed that the accumulation of P25 when coexpressed with NbALY916 was not affected by treatment with MG132 or 3‐MA. NbALY916‐GFP and P25 were coexpressed in *Nicotiana benthamiana* leaves for 48 hr, followed by infiltration of the leaves with 10 mM 3‐MA or 100 μM MG132 for 4 hr. Western blotting was used to determine the levels of P25 and NbALY916 accumulating in these leaves. The asterisks indicating bands of NbALY916‐GFP in western blots. (b) P25 coexpressed with ALY916‐GFP or GFP in the same leaf for 48 hr, followed by 10 mM 3‐MA and 100 μM MG132 for 4 hr. Proteins were detected by western blotting using anti‐P25 or GFP antibody. The P25 protein accumulation was normalized to rubisco and the relative protein levels were calculated in relation to the 1% DMSO‐only treatment. The relative protein levels were calculated by ImageJ

**TABLE S1** Ninety‐eight plant P25‐interacting proteins were collected by coimmunoprecipitation (with green fluorescent protein‐binding magnetic beads and identified by mass spectrometry

**TABLE S2** Primer pairs used in this study

## Data Availability

The data that support the findings of this study are available from the corresponding author upon reasonable request.
