# Supplementary figures and images for: NbALY916 is involved in potato virus X P25‐triggered cell death in Nicotiana benthamiana
Source: Mol Plant Pathol. 2020 Sep 6;21(11):1495–501. doi: 10.1111/mpp.12986 (PMC7549001; doi:10.1111/mpp.12986)

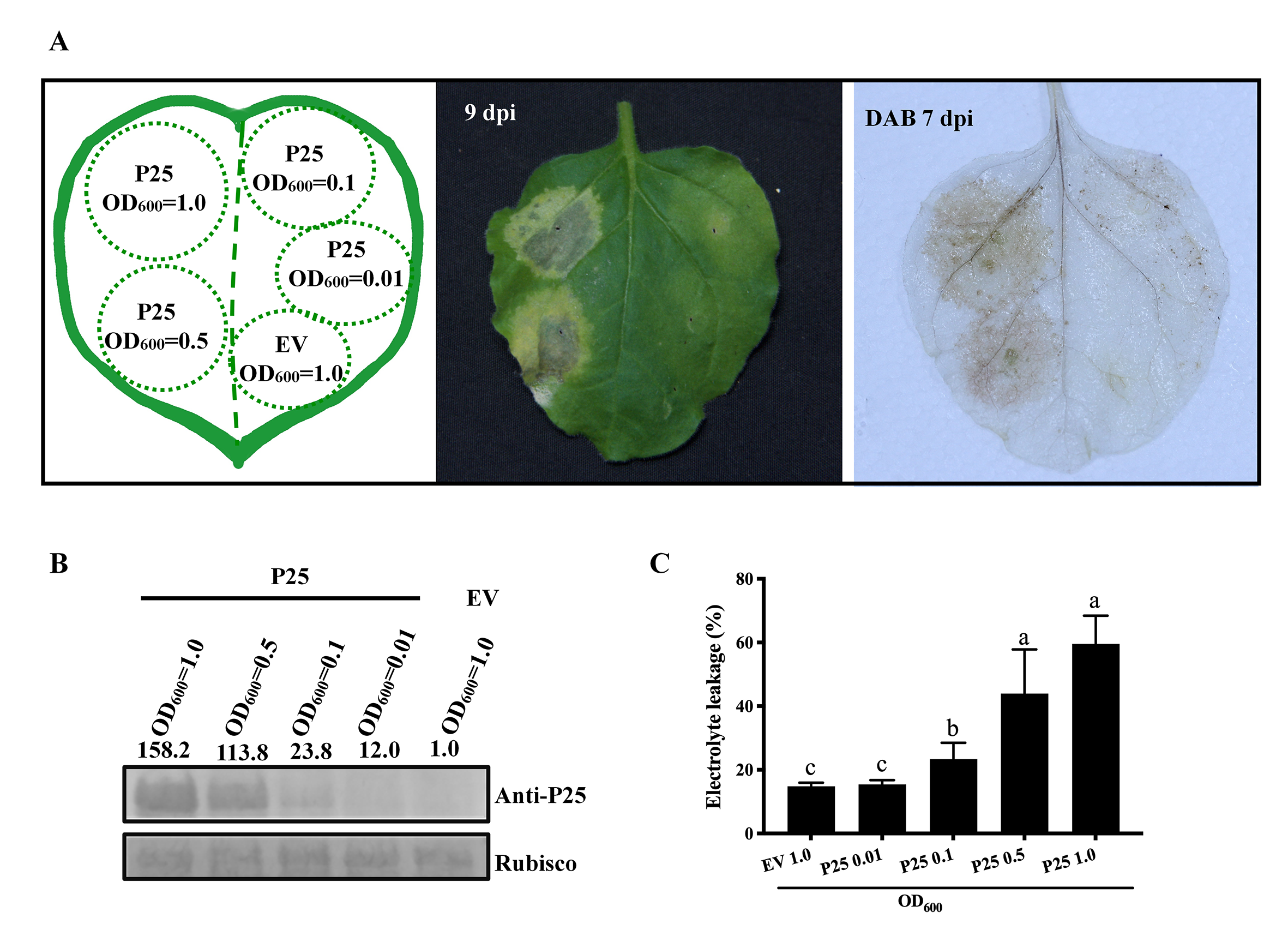

Supplement: Supplementary file 1 — FIGURE S1 PVX P25 induces the accumulation of H2O2 and cell death. (a) Visible necrosis (centre image) and H2O2 accumulation (right image; DAB stained) in Nicotiana benthamiana leaf patches after infiltration with different concentrations (OD600) of agrobacteria harbouring a P25 expression plasmid or empty vector (EV). (b) Western blotting detection of P25 protein in infiltrated patches. The P25 protein accumulation was normalized to RuBisCO and the relative levels were calculated in relation to EV treatment. The relative protein levels were calculated using ImageJ. (c) Leaf discs were excised and assayed for electrolyte leakage. Bars represent the standard errors of the means from three biological repeats, each consisting of six plants. Error bars show SD and the graph represents the combined data from three independent replicates. Letters on the graph denote statistically significant differences (ANOVA, p ≤ .05) [file MPP-21-1495-s001.tif]

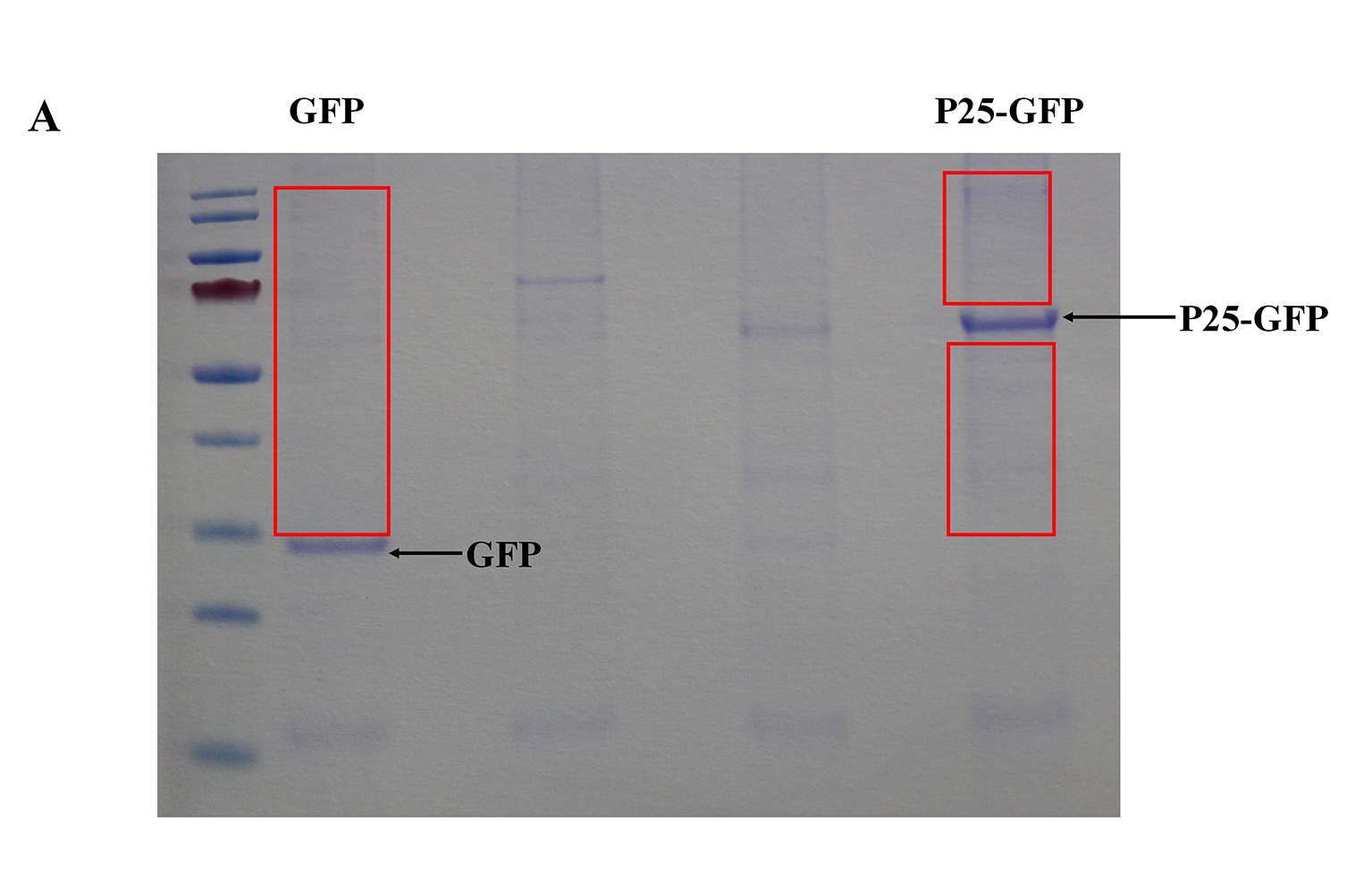

Supplement: Supplementary file 2 — FIGURE S2 Proteins precipitated with transiently expressed green fluorescent protein (GFP) and P25‐GFP by GFP beads. SDS‐PAGE results of precipitated proteins by GFP beads. The GFP lane shows transiently expressing GFP and then precipitation with GFP beads. P25‐GFP lane shows transiently expressing P25‐GFP and then precipitation with GFP beads. The gels in the red boxes were sent for mass spectrum (MS) identification [file MPP-21-1495-s002.tif]

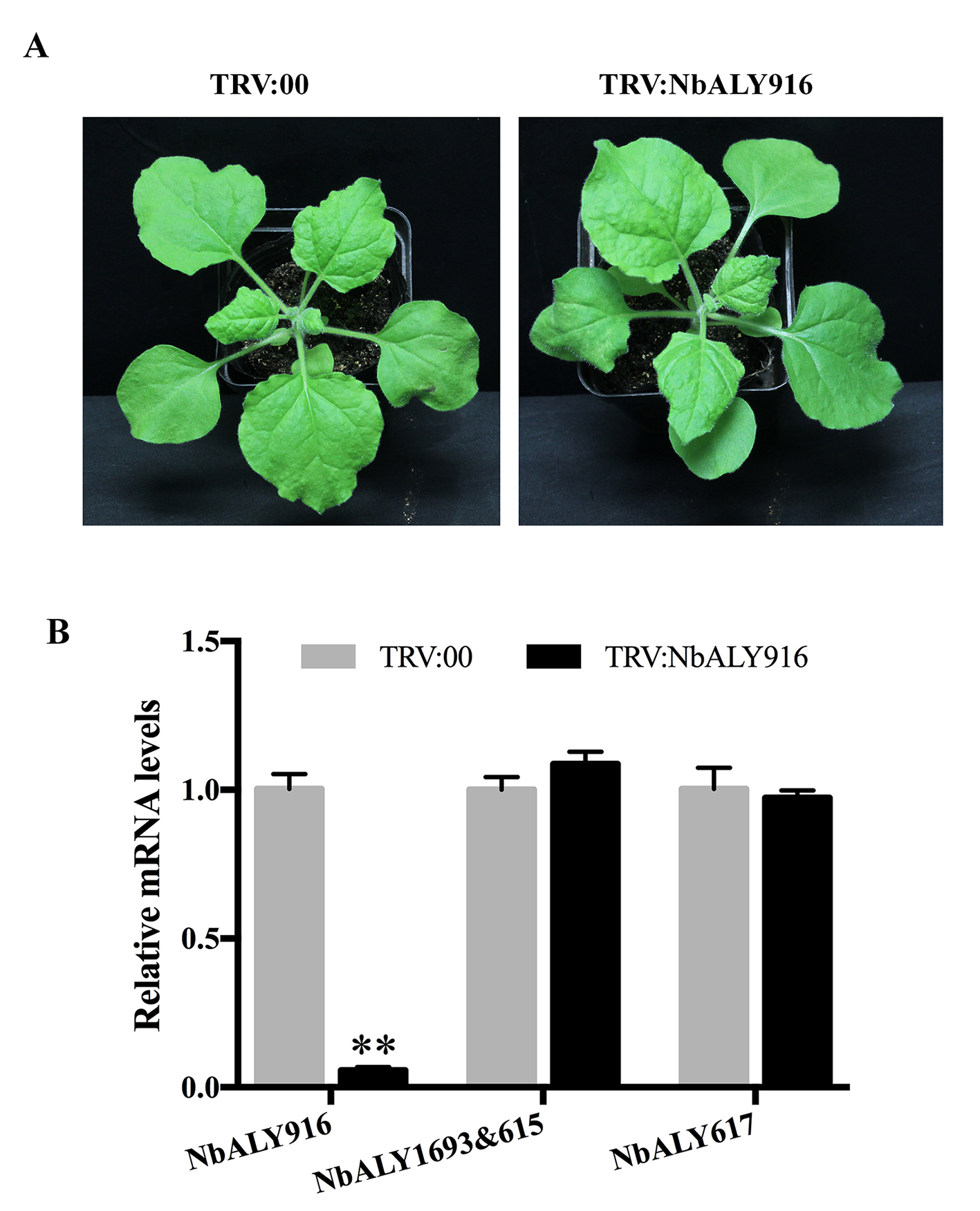

Supplement: Supplementary file 3 — FIGURE S3 Phenotype of TRV‐induced NbALY916 silencing on Nicotiana benthamiana at 10 days postinoculation (dpi). (a) Comparison of infection symptoms caused by TRV:00 and TRV:NbALY916 at 10 dpi. (b) The silencing efficiency of TRV:NbALY916 was measured by quantitative reverse transcription PCR. Primer sequences are listed in Table S2. Bars represent the SEM from three biological repeats. A two‐sample unequal variance directional t test was used to test the significance of the difference (**p < .01) [file MPP-21-1495-s003.tif]

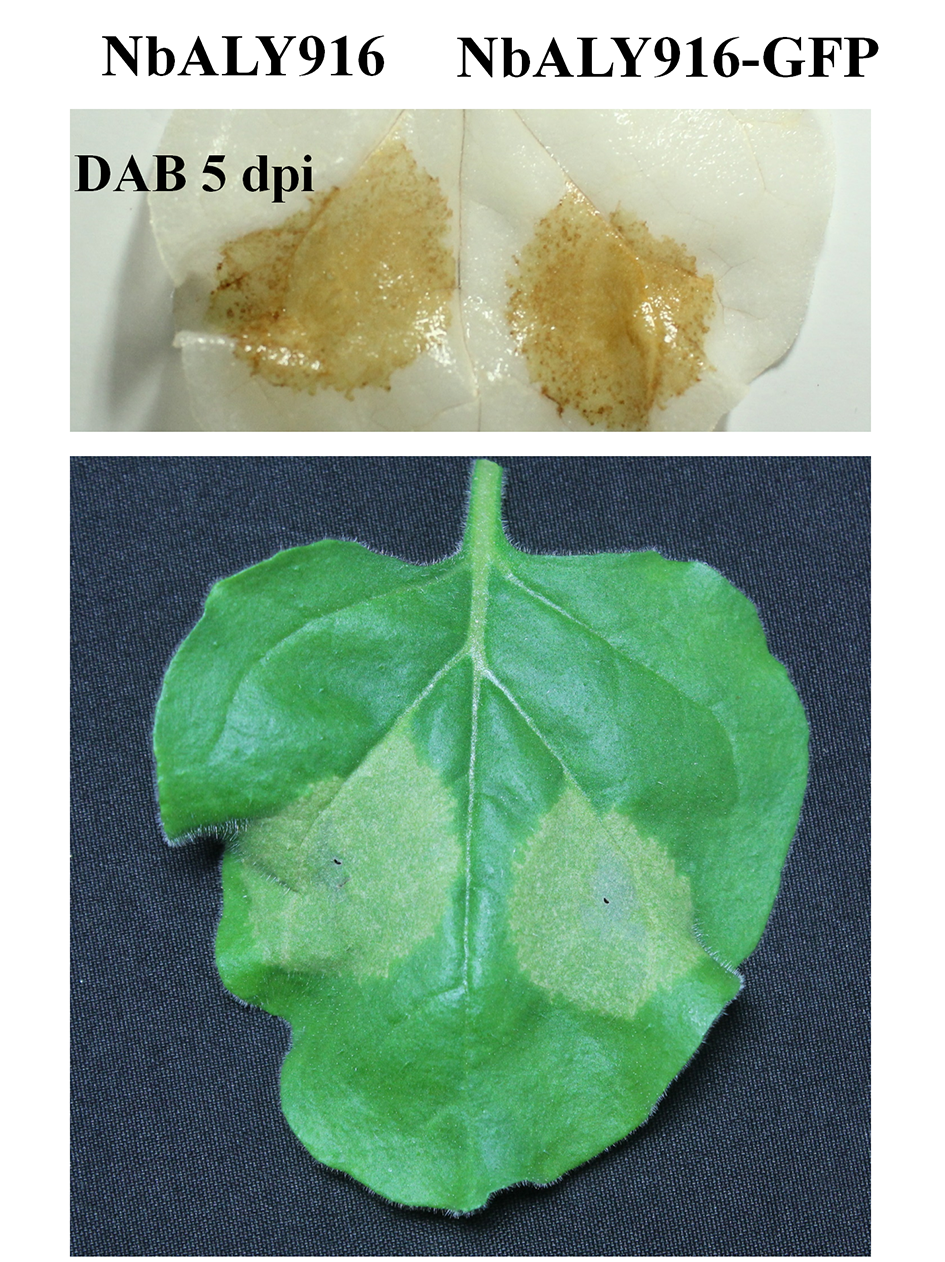

Supplement: Supplementary file 4 — FIGURE S4 Transient overexpression of NbALY916 and NbALY916‐GFP induces cell death. The accumulation of H2O2 (upper panel, DAB stained) and cell death (lower panel, visual symptoms) observed after transient expression of NbALY916‐GFP and NbALY916‐GFP at 5 days postinoculation [file MPP-21-1495-s004.tif]

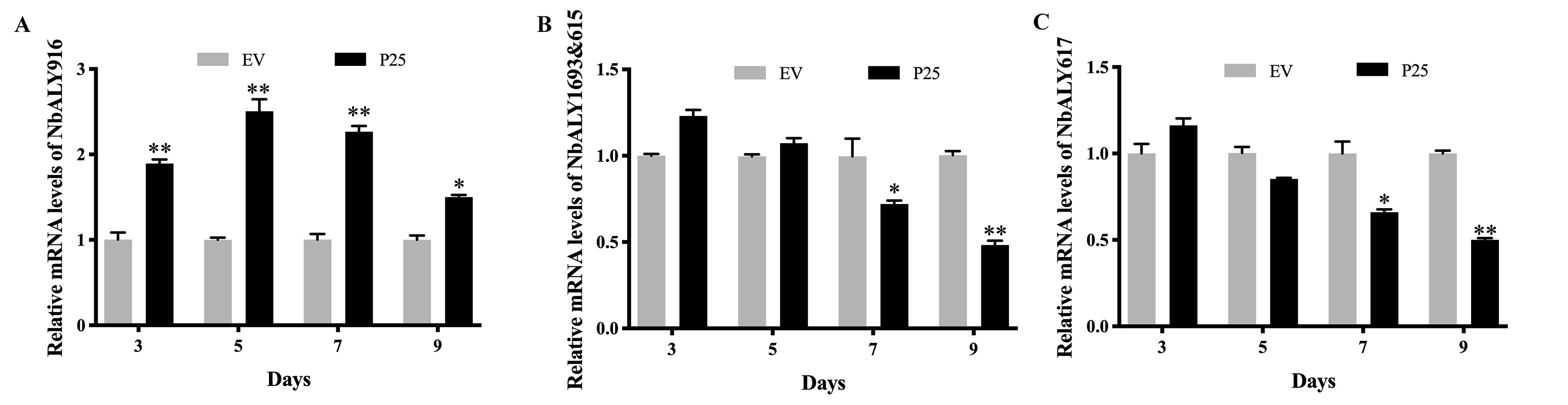

Supplement: Supplementary file 5 — FIGURE S5 P25 induces the expression of NbALY916. Results of quantitative reverse transcription PCR to measure the mRNA levels of NbALY916 (a), NbALY1693 and 615 (b) and NbALY617 (c) in leaves infiltrated with P25 or empty vector (EV). Bars represent the SEM from three biological repeats. A two‐sample unequal variance directional t test was used to test the significance of the difference (*p < .05; **p < .01) [file MPP-21-1495-s005.tif]

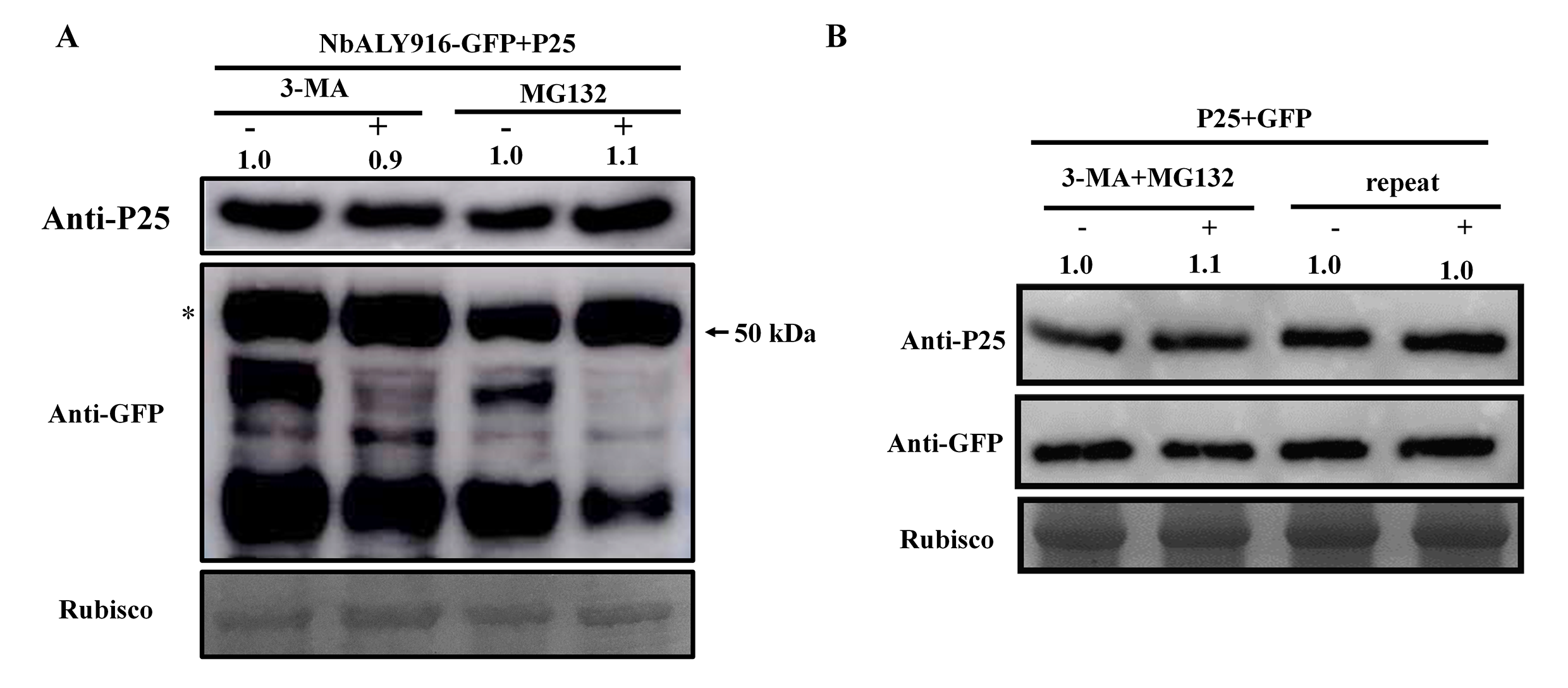

Supplement: Supplementary file 6 — FIGURE S6 The accumulation of P25 with chemical treatment. (a) Western blotting showed that the accumulation of P25 when coexpressed with NbALY916 was not affected by treatment with MG132 or 3‐MA. NbALY916‐GFP and P25 were coexpressed in Nicotiana benthamiana leaves for 48 hr, followed by infiltration of the leaves with 10 mM 3‐MA or 100 μM MG132 for 4 hr. Western blotting was used to determine the levels of P25 and NbALY916 accumulating in these leaves. The asterisks indicating bands of NbALY916‐GFP in western blots. (b) P25 coexpressed with ALY916‐GFP or GFP in the same leaf for 48 hr, followed by 10 mM 3‐MA and 100 μM MG132 for 4 hr. Proteins were detected by western blotting using anti‐P25 or GFP antibody. The P25 protein accumulation was normalized to rubisco and the relative protein levels were calculated in relation to the 1% DMSO‐only treatment. The relative protein levels were calculated by ImageJ [file MPP-21-1495-s006.tif]
