# Supplementary material for: NbALY916 is involved in potato virus X P25‐triggered cell death in Nicotiana benthamiana
Source: Mol Plant Pathol. 2020 Sep 6;21(11):1495–501. doi: 10.1111/mpp.12986 (PMC7549001; doi:10.1111/mpp.12986)
Supplement: Supplementary file 8 — TABLE S2 Primer pairs used in this study [file MPP-21-1495-s008.doc]

**Supplementary Table 1: Primers used in the study**

| Primers | | Use |
| --- | --- | --- |
| ALY916 VIGS-F | 5'- CAGGGATTTCATCTGGATTG -3' | VIGS |
| ALY916 VIGS-R | 5'- TCTATCTTCATTGGCTTCCC -3' | VIGS |
| UBC RT f | 5'- TTTCGGTCCTGATGATACTCCC -3' | qRT-PCR |
| UBC RT r | 5'- CACAGAGCAAAGACTGGATTGA -3' | qRT-PCR |
| EF1A RT f | 5'- GATTGGTGGTATTGGTACTGTC -3' | qRT-PCR |
| EF1A RT r | 5'- AGCTTCGTGGTGCATCTC -3' | qRT-PCR |
| ALY916 RT f | 5'- GCTGCTAATCGTGGTTCA -3' | qRT-PCR |
| ALY916 RT r | 5'- ACGCCATTCTTTCTTCC -3' | qRT-PCR |
| ALY1693&615 RT f | 5'- GGAAAGCCCATGAAAATTGA -3' | qRT-PCR |
| ALY1693&615 RT r | 5'- TCAGTTAGTC TCCATTGCTTC -3' | qRT-PCR |
| ALY617 RT f | 5'- TGCGTCTTCCATATCGACTG -3' | qRT-PCR |
| ALY617 RT r | 5'- TTCAATCTTCATCGGCTTCC -3' | qRT-PCR |
| NbrbohA RT f | 5'- TGTCTTGCTGCTTGTTGGTC -3' | qRT-PCR |
| NbrbohA RT r | 5'- ACTTGTTGCAACGCTCATGT -3' | qRT-PCR |
| NbrbohB RT f | 5'- TAAACAAACGAGGCGGCAAA -3' | qRT-PCR |
| NbrbohB RT r | 5'- GACAGCGACGGAATCTTCAC -3' | qRT-PCR |
| TRV-CP RT f | 5'- ATGGGTGACATGTACGATGA -3' | RT-PCR |
| TRV-CP RT r | 5'- TCAAGGATTAGGACGCACG -3' | RT-PCR |
| probe-PVX CP f | 5'- TGGGACTTAGTCAGACACT -3' | probe |
| probe-PVX CP r | 5'- ACCTCGAGTGACAGCTGC -3' | probe |
